# Ischemic Heart Disease Modifies the Association of Atrial Fibrillation With Mortality in Heart Failure With Reduced Ejection Fraction

**DOI:** 10.1161/JAHA.118.009770

**Published:** 2018-10-03

**Authors:** Ben N. Mercer, Aaron Koshy, Michael Drozd, Andrew M. N. Walker, Peysh A. Patel, Lorraine Kearney, John Gierula, Maria F. Paton, Judith E. Lowry, Mark T. Kearney, Richard M. Cubbon, Klaus K. Witte

**Affiliations:** ^1^ Leeds Institute of Cardiovascular and Metabolic Medicine LIGHT Laboratories The University of Leeds United Kingdom

**Keywords:** atrial fibrillation, heart failure, hospitalization, ischemic, mortality, Atrial Fibrillation, Heart Failure, Mortality/Survival

## Abstract

**Background:**

The CASTLE‐AF (Catheter Ablation versus Standard Conventional Therapy in Patients With Left Ventricular Dysfunction and Atrial Fibrillation) trial recently reported that catheter ablation of atrial fibrillation (AF) improves survival in heart failure (HF) with reduced ejection fraction (HFrEF). However, established AF was not associated with mortality in trials of contemporary HFrEF pharmacotherapies. We investigated whether HFrEF pathogenesis may influence the conclusions of studies evaluating the prognostic impact of AF.

**Methods and Results:**

Using a prospective cohort study of 791 patients with HFrEF, with AF determined using 24‐hour ambulatory ECG monitoring, univariable and multivariable Cox regression analyses were used to define the association between AF and mode‐specific mortality (mean follow‐up of 5.4 years). One‐year HF‐related hospitalization was assessed with binary logistic regression analysis. One‐year cardiac remodeling was assessed in a subgroup (n=378) using echocardiography. AF was present in 28.2% of patients, with 9.4% of these being paroxysmal. While AF was associated with increased risk of all‐cause mortality (hazard ratio, 1.27; 95% confidence interval 1.03–1.57), with diverging survival curves after 1 year of follow‐up, this association was lost in age‐sex–adjusted analyses. However, AF was associated with increased risk of age‐sex–adjusted all‐cause mortality in people with ischemic pathogenesis, with a statistically significant interaction between pathogenesis and AF. This was predominantly attributed to progressive HF deaths. After 1 year, HF hospitalization and cardiac remodeling were not associated with AF, even in people with ischemic pathogenesis.

**Conclusions:**

AF is associated with increased risk of death in HFrEF of ischemic pathogenesis, predominantly due to progressive HF deaths during long‐term follow‐up. HFrEF pathogenesis should be considered in trial design and interpretation.

**Clinical Perspective**

**What Is New?**

- Overall, atrial fibrillation (AF) is not associated with age‐sex–adjusted all‐cause or mode‐specific mortality in patients with heart failure and a reduced ejection fraction (HFrEF) receiving contemporary therapy.
- However, AF outcomes significantly interact with HFrEF pathogenesis, such that only patients with AF and ischemic HFrEF experience increased risk of death.
- AF is not associated with risk of heart failure hospitalization or cardiac remodeling after 1 year of follow‐up, even in ischemic HFrEF.

**What Are the Clinical Implications?**

- Clinical trials of AF therapies in HFrEF should focus on patients with ischemic pathogenesis and have long‐term follow‐up, to attempt to address this question of how the increased mortality in this subgroup of patients with HF could be reduced.

## Introduction

Atrial fibrillation (AF) is the most common arrhythmia encountered in patients with chronic heart failure (HF),^1^ and its prevalence increases as symptoms progress, approaching 50% in patients with New York Heart Association class IV dyspnea.^2^ The literature describing the association of AF with adverse outcomes in people with HF and reduced ejection fraction (HFrEF) is conflicting, with only some studies identifying AF as an independent risk factor for mortality and morbidity.^2^, ^3^ Other studies have reported that when analyses are adjusted for common confounding factors, such as demographics, AF is no longer an independent predictor of death.^4^, ^5^ A meta‐analysis including studies from 1996 to 2008 found that, after adjustment for confounding factors, AF was associated with increased all‐cause mortality.^6^ This analysis largely included participants of randomized controlled trials of treatments that are now considered standard in the management of HFrEF, and device therapy was not routine. More contemporary data from a post hoc analysis of the PARADIGM‐HF (Prospective Comparison of ARNI With ACEI to Determine Impact on Global Mortality and Morbidity in Heart Failure) and ATMOSPHERE (Aliskiren Trial to Minimize Outcomes in Patients With Heart Failure) trials found that paroxysmal AF (pAF), but not persistent/permanent AF, was associated with increased composite risk of cardiovascular death or HF hospitalization. Neither pAF nor persistent/permanent AF was associated with increased all‐cause mortality, and no subgroup analyses of ischemic pathogenesis were conducted.^7^ Some reports suggest that AF is associated with increased mortality in patients with ischemic HF, but include patients with HF and preserved ejection fraction (HFpEF) and apply outdated therapeutic strategies.^8^, ^9^ It is therefore unclear whether there is an adverse interaction between AF and ischemic pathogenesis in patients receiving contemporary HFpEF management.

Against the backdrop of these conflicting data, interventional therapies for AF are increasingly applied in people with HFrEF, following reports of improvements in left ventricular (LV) ejection fraction and functional capacity.^10^ Moreover, recently published data have suggested a reduction in mortality and HF hospitalization with the use of catheter ablation for paroxysmal or persistent AF in the setting of HFrEF.^11^ However, the discrepancy with the described analysis of the PARADIGM‐HF and ATMOSPHERE trials^7^ emphasizes the need to understand which patients with HFrEF experience an increased risk of adverse events associated with AF, in order to guide clinical practice and research. We have explored the association between AF and mode‐specific mortality, hospitalization, and cardiac remodeling in a prospectively recruited HFrEF cohort, particularly focusing on the interaction between AF and ischemic pathogenesis.

## Methods

The data, analytic methods, and study materials will not be made available to other researchers for purposes of reproducing the results or replicating the procedure since the complete study data set contains potentially identifying data; however, data will be made available by the corresponding author to other researchers who have appropriate ethical approval and data protection arrangements.

We conducted a prospective multicenter cohort study of 1091 patients, aiming to define risk factors for adverse outcomes in people with HFrEF receiving contemporary evidence‐based care.^12^ All patients were adults (age ≥18 years) with stable signs and symptoms of chronic HF for at least 3 months, had LV ejection fraction ≤45% on 2‐dimensional transthoracic echocardiography, and were recruited between June 2006 and December 2011. Leeds West Research Ethics Committee provided ethical approval, and all recruits provided written informed consent, in accordance with the Declaration of Helsinki. This analysis is restricted to the 791 patients with complete ambulatory 24‐hour ECG data to ensure systematic assessment of cardiac rhythm.^12^

As previously described,^12^ details of medical history and drug history were collected at recruitment. Symptomatic status was defined using New York Heart Association classification. Transthoracic echocardiography was performed according to British Society of Echocardiography recommendations.^13^ Venous blood was collected for measurement of electrolyte concentrations, assessment of renal function, and hematological parameters, which were performed in the local hospital chemical pathology laboratories. Estimated glomerular filtration rate was calculated using the Modification of Diet in Renal Disease method.^14^ Resting heart rate was measured using 12‐lead ECG. Use and dosing of diuretic therapy, angiotensin‐converting enzyme inhibitors, angiotensin receptor blockers, and β‐blockers were collected at study recruitment. The prescribed daily doses of β‐blockers were expressed relative to the maximal licensed dose of bisoprolol and diuretic dose was normalized to furosemide.^15^ Receipt of cardiac resynchronization therapy or implantable cardioverter‐defibrillator (ICD) was defined 6 months after recruitment to account for device implantation shortly after referral to the service. In a subset of 408 patients, clinical review was repeated ≈1 year later to document changes in symptomatic status and LV dimensions (remodeling). This subset represents all patients within the first cohort of 628 participants who were alive and willing to attend a study follow‐up visit, as previously described^16^ of whom 378 had 24‐hour ambulatory ECG data, so were included in this analysis.

### Assessment of AF

During normal, unrestricted, out‐of‐hospital activity, 24‐hour ambulatory 3‐lead ECGs (Lifecard CF, Spacelabs Healthcare) were obtained. Patients with atrial flutter were included in the AF group, given the frequent co‐occurrence of these rhythms. Patients with AF that was not sustained throughout monitoring were labelled as having pAF. Since monitoring lasted for 24 hours, no further classification of persistent or permanent AF was made. Maximum and minimum ambulatory heart rate was recorded as described.^12^

### Mortality and Hospitalization Assessment

All patients were registered with the UK Office of Population Censuses and Surveys, which provided details of death. Censorship took place on May 8, 2016. Classification criteria for the mode of death were defined before the study commenced, based on previous publications.^17^ At least 2 senior physicians reviewed each death certificate and gathered data as required from autopsy reports, hospital notes, and primary care records. Mode of death was classified as: (1) sudden cardiac, if it occurred within 1 hour of a change in symptoms or during sleep or while the patient was unobserved (defibrillator therapies were not included as a proxy); (2) progressive HF, if death occurred after a documented period of symptomatic or hemodynamic deterioration; (3) other cardiovascular death, if not occurring suddenly or in association with progression of HF (eg, cerebrovascular accident); (4) noncardiovascular death; and (5) unclassifiable, where insufficient information was available to reach a firm conclusion. HF‐related hospitalization was assessed as described,^12^ using institutional clinical event databases detailing all admissions in recruiting centers. Details of all nonelective hospitalizations were also collected. Events were assessed during the first year of recruitment and analyzed as a binary outcome.

### Statistical Analysis

All analyses were conducted with SPSS version 23 (IBM). Continuous data are displayed as means (SEM), and categorical data are displayed as percentages (number). Normality of distribution was confirmed on skewness testing. Continuous data were compared with unpaired or paired Student *t* tests, as appropriate, and categorical data with χ^2^ tests. Cox proportional hazards regression was used in univariate and multivariate mortality analyses, and binary logistic regression analysis was used for hospitalization analyses. Assessment of partial residuals was used to confirm no deviation from the assumption of proportional hazards. Statistical significance was defined as *P*<0.05.

## Results

Of the 791 patients included in this study, AF was present during ambulatory ECG monitoring in 223 (28.2%), and this was paroxysmal in 21 (9.4%) of those; baseline characteristics of patients with and without any AF are presented in Table 1. Patients with AF were older and less likely to have HF due to ischemic pathogenesis but had similar LV ejection fraction and showed only a trend toward worse symptoms assessed by New York Heart Association class. Patients with AF had a higher resting heart rate on 12‐lead ECG, along with higher minimum and maximum ambulatory heart rates. The only significant differences in HF pharmacotherapy were higher diuretic doses and greater digoxin use in patients with AF, while anticoagulation was also more commonly prescribed to those with AF. There were similar rates of cardiac resynchronization therapy implantation in each group, but patients with AF were less likely to have an ICD. Lower rates of ICD implantation in patients with AF may be related to their greater age and comorbidity.

**Table 1:** Baseline Patient Characteristics

	No AF	AF	*P* Value
Age, y	66.5 (0.5)	71.2 (0.7)	<0.001
Men	72.4 (411)	77.1 (172)	0.17
Ischemic pathogenesis	66.7 (379)	55.2 (123)	0.002
Diabetes mellitus	25.7 (146)	26 (58)	0.93
COPD	13.7 (78)	13.9 (31)	0.95
PPM/ICD/CRT*	33.3 (189)	28.7 (64)	0.22
ICD	14.8 (84)	5.4 (12)	<0.001
CRT	25 (142)	24.2 (54)	0.82
NYHA class 3/4	31.8 (180)	39 (87)	0.054
Warfarin	18.6 (105)	62.6 (139)	<0.001
ACEI/ARB	89.2 (504)	85.6 (190)	0.16
β‐Blocker	79.8 (451)	77.5 (172)	0.47
MRA	38.6 (218)	45 (100)	0.096
Digoxin	12.9 (73)	44.4 (99)	<0.001
Amiodarone	9.9 (56)	7.6 (17)	0.33
Systolic BP, mm Hg	122.3 (0.9)	118.9 (1.4)	0.043
Resting HR on 12‐lead ECG, bpm	72.2 (0.8)	78.5 (1.5)	<0.001
QRS interval, ms	122.9 (1.3)	119.6 (2.3)	0.2
Hemoglobin, g/dL	13.5 (0.1)	13.9 (0.1)	0.013
Sodium, mmol/L	139.3 (0.1)	139.5 (0.2)	0.43
eGFR, mL/min per 1.73 m^2^	55.8 (0.7)	52.8 (1.1)	0.029
LVEF, %	32 (0.4)	32.3 (0.6)	0.71
Minimum ambulatory HR, bpm	56.5 (0.5)	59.3 (1)	0.009
Maximum ambulatory HR, bpm	102.6 (0.8)	118.9 (1.8)	<0.001
Ramipril equivalent dose, mg/d	5 (0.2)	4.7 (0.2)	0.17
Bisoprolol equivalent dose, mg/d	3.4 (0.1)	3.7 (0.2)	0.23
Furosemide equivalent dose, mg/d	50.5 (2.2)	59.5 (3.4)	0.026

Continuous data are presented as mean (SEM) and compared with Student *t* tests. ACEI indicates angiotensin‐converting enzyme inhibitor; AF, atrial fibrillation; ARB, angiotensin receptor blocker; BP, blood pressure; bpm, beats per minute; COPD, chronic obstructive pulmonary disease; CRT, cardiac resynchronization therapy; eGFR, estimated glomerular filtration rate; HR, heart rate; ICD, implantable cardioverter‐defibrillator; LVEF, left ventricular ejection fraction; MRA, mineralocorticoid receptor antagonist; NYHA, New York Heart Association; PPM, permanent pacemaker.

### Total and Mode‐Specific Mortality

**Figure 1:**
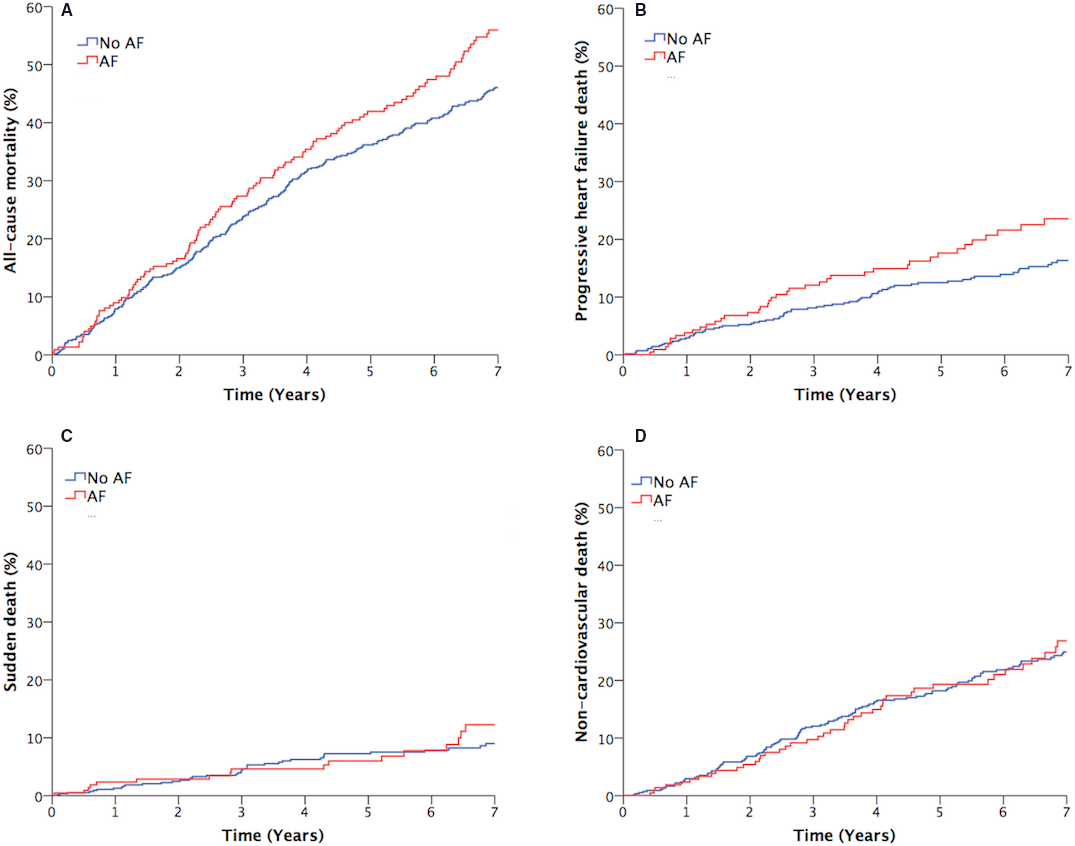
Unadjusted all‐cause and mode‐specific mortality. Kaplan‐Meier curves illustrating (A) all‐cause, (B) progressive heart failure, (C) sudden, and (D) noncardiovascular mortality. AF indicates atrial fibrillation.

After a mean follow‐up period of 5.4 years, 407 deaths had occurred, of which 232 were cardiovascular (including 127 progressive HF deaths and 59 sudden deaths) and 175 were noncardiovascular. The presence of AF was associated with increased risk of all‐cause mortality (1.27; 95% confidence interval, 1.03–1.57 [*P*<0.05]), primarily driven by an increase in progressive HF death (1.49; 95% confidence interval, 1.03–2.15 [*P*<0.05]) (Figure 1). However, these associations were lost in age‐sex–adjusted analyses (Table 2), suggesting that the older age and male preponderance of patients with AF may account for much of their increased risk of death in unadjusted analyses. If differences in the treatment of patients with AF (ICD provision, diuretic dose, warfarin use, and digoxin use) were also included in age‐sex–adjusted analyses, the neutral association of AF with mortality was unchanged (Table 2). Similarly, if resting heart rate was added to age‐sex–adjusted analyses, the neutral association of AF with mortality was unchanged (Table 2). However, when differences in the comorbidity of patients with AF (ischemic pathogenesis, hemoglobin, and estimated glomerular filtration rate) were included in age‐sex–adjusted analyses, the detrimental association of AF with cardiovascular and progressive HF death returned (Table 2).

**Table 2:** All‐Cause and Mode‐Specific Mortality Analyses

Model	Mode of Death				
	All‐Cause	Cardiovascular	Progressive HF	Sudden	Noncardiovascular
Unadjusted	1.27 (1.03–1.57)*	1.49 (1.12–1.97)*	1.49 (1.03–2.15)*	1.23 (0.7–2.14)	1.03 (0.73–1.43)
Age‐sex	1.04 (0.84–1.28)	1.23 (0.92–1.63)	1.19 (0.83–1.72)	1.16 (0.64–1.96)	0.84 (0.6–1.17)
Age‐sex and treatment†	1.03 (0.81–1.320	1.13 (0.81–1.58)	1.04 (0.68–1.59)	1.01 (0.52–1.97)	0.91 (0.62–1.34)
Age‐sex and comorbidity‡	1.23 (0.99–1.54)	1.49 (1.1–2.01)*	1.48 (1–2.2)*	1.37 (0.76–2.49)	1.05 (0.74–1.49)
Age‐sex and HR§	1.04 (0.83–1.32)	1.28 (0.94–1.74)	1.32 (0.89–1.98)	1.13 (0.63–2.05)	0.83 (0.57–1.19)

Hazard ratios with 95% confidence intervals indicating the risk of all‐cause or mode‐specific death in people with atrial fibrillation. HF indicates heart failure.

*P<0.05; ^†^age, sex, furosemide dose, implantable cardioverter‐defibrillator, warfarin, and digoxin; ^‡^age, sex, ischemic pathogenesis, hemoglobin, and estimated glomerular filtration rate; ^§^age, sex, and resting heart rate (HR).

**Figure 2:**
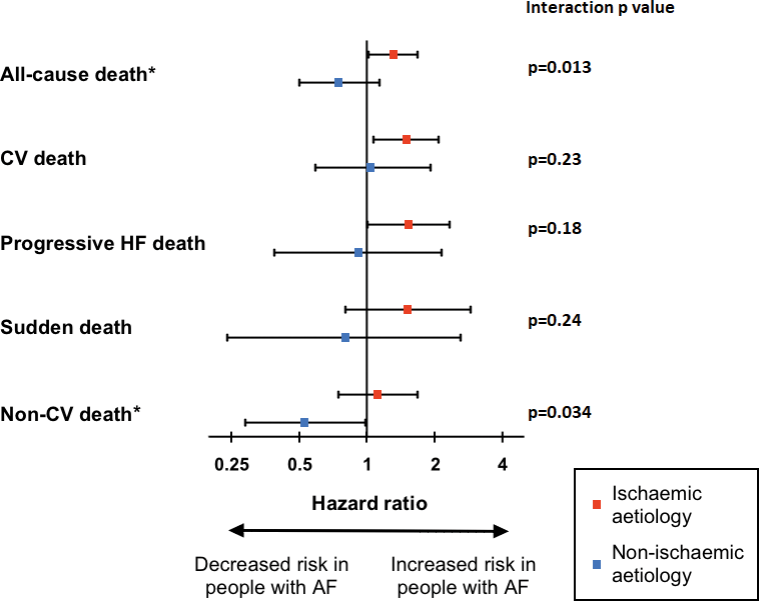
Age‐sex–adjusted all‐cause and mode‐specific mortality in subgroups with ischemic and nonischemic heart failure (HF). Forest plot demonstrating hazard ratios (with 95% confidence intervals) for all‐cause and mode‐specific mortality in patients with ischemic and nonischemic causes. CV indicates cardiovascular. *Statistically significant interaction between pathogenesis and atrial fibrillation (AF; indicating confidence intervals are different with *P*<0.05).

In view of the significant association of AF with cardiovascular death in our age‐sex– and comorbidity‐adjusted analyses, and published data linking AF with adverse outcomes in ischemic heart disease (IHD), we assessed age‐sex–adjusted mortality in subgroups with ischemic and nonischemic HF. AF was associated with increased risk of all‐cause mortality in people with ischemic pathogenesis, but not in those with nonischemic pathogenesis, with a statistically significant age‐sex–adjusted interaction between pathogenesis and AF (Figure 2). Sensitivity analyses excluding patients with pAF, or those prescribed amiodarone, revealed no change in this interaction between AF and ischemic pathogenesis (*P*=0.005 and *P*=0.017, respectively), suggesting that these factors are unlikely in isolation to explain this phenomenon. Further exploration of mode‐specific mortality revealed a similar pattern for cardiovascular, progressive HF, and sudden death, although no significant interaction was present in any of these analyses. However, the risk of noncardiovascular death was significantly reduced in people with nonischemic (but not ischemic) HF with a significant interaction between pathogenesis and AF. In order to explore these observations further, we defined patient characteristics according to the presence or absence of AF in subgroups with ischemic and nonischemic pathogenesis (Table 3). This showed that patients with ischemic HF and AF were prescribed significantly higher doses of loop diuretics and were more likely to receive a mineralocorticoid receptor antagonist. This was not the case in people with AF and nonischemic HF. This is congruent with the higher rates of progressive HF death in patients with AF in the context of ischemic HF.

**Table 3:** Baseline Patient Characteristics According to HF Pathogenesis

	Nonischemic Subgroup			Ischemic Subgroup		
	No AF (n=189)	AF (n=100)	*P* Value	No AF (n=379)	AF (n=123)	*P* Value
Age, y	59.6 (1.0)	67.4 (1.2)	<0.001	70 (0.5)	74.4 (0.8)	<0.001
Men	64 (121)	72 (72)	0.17	76.5 (290)	81.3 (100)	0.27
Diabetes mellitus	13.8 (26)	17 (17)	0.46	31.7 (120)	33.3 (41)	0.73
COPD	9.5 (18)	8 (8)	0.67	15.8 (60)	18.7 (23)	0.46
PPM/ICD/CRT	29.6 (56)	18 (18)	0.031	35.1 (133)	37.4 (46)	0.64
ICD	8.5 (16)	2 (2)	0.03	17.9 (68)	8.1 (10)	0.009
CRT	25.9 (49)	15 (15)	0.033	24.5 (93)	31.7 (39)	0.12
NYHA class 3/4	25.9 (49)	29 (29)	0.58	34.7 (131)	47.2 (58)	0.014
Warfarin	19.7 (37)	67 (67)	<0.001	18.0 (68)	59.0 (72)	<0.001
ACEI/ARB	91.5 (172)	84 (84)	0.054	88.1 (332)	86.9 (106)	0.73
β‐Blocker	79.8 (150)	83 (83)	0.51	79.8 (301)	73.0 (89)	0.11
MRA	36.2 (68)	38 (38)	0.76	39.8 (150)	50.8 (62)	0.032
Digoxin	14.8 (28)	53 (53)	<0.001	11.9 (45)	37.4 (46)	<0.001
Amiodarone	7.9 (15)	10 (10)	0.55	10.8 (41)	5.7 (7)	0.093
Systolic BP, mm Hg	121.0 (1.6)	118.6 (2.1)	0.38	122.9 (1.2)	119.2 (1.8)	0.079
Resting HR on 12‐lead ECG, bpm	77.0 (1.5)	84.5 (2.2)	0.004	70.0 (0.9)	73.5 (1.8)	0.082
QRS interval, ms	123.6 (2.5)	111.0 (3.4)	0.003	122.6 (1.6)	126.7 (2.9)	0.21
Hemoglobin, g/dL	13.9 (0.1)	14.3 (0.2)	0.091	13.3 (0.1)	13.5 (0.2)	0.23
Sodium, mmol/L	139.8 (0.2)	139.7 (0.3)	0.92	139.0 (0.2)	139.3 (0.3)	0.5
eGFR, mL/min per 1.73 m^2^	61.6 (1.3)	58.1 (1.6)	0.093	52.9 (0.9)	48.5 (1.5)	0.013
LVEF, %	29.3 (0.7)	32.5 (0.9)	0.01	33.4 (0.4)	32.2 (0.8)	0.16
Minimum ambulatory HR, bpm	58.6 (0.9)	62.7 (1.5)	0.019	55.5 (0.5)	56.6 (1.2)	0.38
Maximum ambulatory HR, bpm	111.2 (1.4)	131.1 (2.8)	<0.001	98.3 (0.9)	109.0 (1.9)	<0.001
Ramipril equivalent dose, mg/d	5.0 (0.2)	4.5 (0.4)	0.19	5.1 (0.2)	4.8 (0.3)	0.54
Bisoprolol equivalent dose, mg/d	3.1 (0.2)	4.3 (0.4)	0.005	3.6 (0.2)	3.3 (0.3)	0.39
Furosemide equivalent dose, mg/d	46.9 (3.7)	44.2 (4.3)	0.65	52.3 (2.7)	72.1 (4.7)	<0.001

ACEI indicates angiotensin‐converting enzyme inhibitor; AF, atrial fibrillation; ARB, angiotensin receptor blocker; BP, blood pressure; bpm, beats per minute; COPD, chronic obstructive pulmonary disease; CRT, cardiac resynchronization therapy; eGFR, estimated glomerular filtration rate; HF, heart failure; HR, heart rate; ICD, implantable cardioverter‐defibrillator; LVEF, left ventricular ejection fraction; MRA, mineralocorticoid receptor antagonist; NYHA, New York Heart Association; PPM, permanent pacemaker.

### Hospitalization, Cardiac Remodeling, and Symptomatic Deterioration After 1 Year

After 1 year of follow‐up, there were 103 cardiovascular hospitalizations, of which 46 were caused by worsening HF. The presence of AF was not associated with the age‐sex–adjusted odds of cardiovascular (0.67; 95% confidence interval, 0.4–1.11 [*P*=0.12]) or HF‐specific (0.92; 95% confidence interval, 0.46–1.83; [*P*=0.81]) hospitalization. In keeping with this, there were also no significant differences in cardiac remodeling, as measured by the change in LV end‐diastolic diameter, LV end‐systolic diameter, and LV ejection fraction after 1 year (Table 4). We also noted no difference in the prevalence of declining New York Heart Association class 1 year after recruitment. Notably, the observed neutral association of AF with hospitalization, LV remodeling, and symptomatic deterioration was unchanged in analyses restricted to patients with ischemic or nonischemic HF.

**Table 4:** Change in Clinical Parameters After 1 Year

	No AF (n=270)	AF (n=108)	*P* Value
Change in LVEDD, mm	−2.4 (0.5)	−1.5 (0.7)	0.32
Change in LVESD, mm	−3.6 (0.6)	−3.4 (0.8)	0.84
Change in LVEF	7 (0.7)	6.8 (1.1)	0.86
Decline in NYHA class	15.9 (43)	9.3 (10)	0.092

Data are presented as mean (SEM) or percentage (number). AF indicates atrial fibrillation; LVEDD, left ventricular end‐diastolic dimension; LVEF, left ventricular ejection fraction; LVESD, left ventricular end‐systolic dimension; NYHA, New York Heart Association.

## Discussion

Our analysis of a large HFrEF cohort with robust baseline cardiac rhythm assessment and long‐term mode‐specific mortality data provides important new insights into subgroups at risk for adverse cardiovascular events associated with AF. While AF was crudely associated with increased risk of all‐cause and cardiovascular death, simple age‐sex adjustment abrogated these findings. However, further analyses revealed a significant interaction with ischemic pathogenesis, such that both all‐cause and cardiovascular death were higher in people with ischemic HF. Notably, survival curves began to diverge beyond 1 year of follow‐up, potentially explaining our observation that HF hospitalization and adverse cardiac remodeling were not more likely in people with AF, even in analyses restricted to ischemic HF. Our data may offer some explanation for the conflicting literature on the adverse prognostic association of AF and suggest that ongoing trials of AF interventions should focus on populations with ischemic HF and also apply extended follow‐up periods.

### AF and Mortality in Populations With HF: Insights From Conflicting Data

A meta‐analysis of studies published from 1996 to 2008 reported that AF was associated with increased all‐cause mortality (hazard ratio [HR], 1.4) in those with HF, a finding that persisted even after adjustment for confounding factors.^6^ However, this analysis included studies with relatively low use of contemporary therapies, such as β‐blockers and angiotensin‐converting enzyme inhibitors (prescribed in ≈24% and 65% of patients, respectively). Furthermore, mineralocorticoid receptor antagonist and cardiac resynchronization therapy/ICD use was not reported. In contrast, post hoc analysis of the most contemporary randomized controlled trial data suggested that only pAF was associated with increased risk of composite cardiovascular death or HF hospitalization; however, this analysis of the PARADIGM‐HF and ATMOSPHERE trials indicated that AF of any type was not associated with increased risk of all‐cause mortality.^7^ Again, and in keeping with our own findings, the HF‐ACTION (Heart Failure: A Controlled Trial Investigating Outcomes of Exercise Training) study concluded that AF is not an independent risk factor for increased mortality or hospitalization in patients with HFrEF.^18^ These data markedly contrast with those of the recently published CASTLE‐AF (Catheter Ablation versus Standard Conventional Therapy in Patients With Left Ventricular Dysfunction and Atrial Fibrillation) trial, which reported a 38% reduction in all‐cause mortality or HF hospitalization in patients receiving catheter ablation.^11^ Such remarkable data must be placed in the context of the relatively small (and lower‐than‐planned) sample of highly selected patients receiving an open‐label intervention. However, the trial does raise important questions about targeting specific subgroups at the greatest risk of adverse events associated with AF. Their inclusion of patients with pAF is supported by the analysis of the PARADIGM‐HF and ATMOSPHERE trials, yet the subgroup analysis of the CASTLE‐AF trial showed no interaction with paroxysmal/persistent AF. Our own analysis shows that an ischemic cause should also be considered a risk factor for AF‐associated mortality, and CASTLE‐AF showed a slightly larger reduction in primary outcome in patients with ischemic HF (40% versus 26%), although no significant interaction was present. Notably, survival curves only began to diverge after 3 years of follow‐up in the CASTLE‐AF trial, somewhat mirroring our own observations.

### AF and HF of Ischemic Pathogenesis

In 2006, Pedersen et al^9^ reported that AF was only associated with increased all‐cause mortality in patients with a history of IHD. However, only ≈50% of these patients were prescribed an angiotensin‐converting enzyme inhibitor, as patients were recruited in 1993–1995, predating the introduction of standard therapies including β‐blockers, mineralocorticoid receptor antagonists, and complex devices. Raunsø et al^8^ also observed in a cohort of patients with mixed HFrEF and HFpEF recruited between 2001 and 2004 that chronic AF was associated with increased risk of death in the subgroup with IHD. These data are affected by similar limitations. Our data substantially expand these observations by showing relevance in the context of contemporary HFrEF therapies and providing detailed mode‐specific outcome data, including for cardiac remodeling. Although the observational nature of our study prevents a thorough assessment of the mechanisms underpinning our findings, our data suggest that AF may aggravate the HF syndrome to a greater extent in people with ischemic HF. Specifically, we noted greater loop diuretic dose and use of mineralocorticoid receptor antagonists in patients with ischemic HF and AF, along with higher rates of progressive HF death. Notably, the age‐sex–adjusted interaction between AF and ischemic pathogenesis in all‐cause mortality analyses was lost (*P*=0.06) when further adjusting for diuretic dose, implying differences in diuretic dose to explain at least a proportion of the interaction. There are also animal data demonstrating that AF can increase oxidative stress, ventricular ischemia, and endothelial dysfunction,^19^ while AF decreases myocardial blood flow in humans.^20^ One could speculate that these pathophysiological effects may be more important in patients with AF and HF due to IHD, although we have no data as an objective measure of myocardial ischemia in our cohort. It will be important for future studies to explore these observations and those made by our study.

### Study Limitations

It is important to acknowledge some limitations of our study. First, observational studies cannot causally implicate AF, but even randomized controlled trials of AF therapies cannot fully address this question, as they are not uniformly effective and suffer from crossover. Notably, prospective cohort studies may also allow assessment of less selected populations than post hoc analyses of randomized controlled trials. Second, the manner in which we systematically determined the presence of AF is a potential limitation, as this is not necessarily practical in real‐world care. It is also important to acknowledge that longer periods of monitoring may have identified more cases of AF. However, this factor is not one limited to our study, and lack of interval monitoring of heart rhythm is a common limitation in much of the literature that investigates the prognostic impact of AF in patients with HF. Indeed, the majority of published studies classify AF based on medical history or a 12‐lead ECG alone. Finally, the relatively low proportion of pAF means that we do not have the statistical power to assess the prognosis of this important subgroup.

## Conclusions

Our study suggests that AF is associated with increased risk of all‐cause and cardiovascular death, but only in patients with HFrEF caused by IHD, and this risk only manifests during longer‐term follow‐up. These data suggest that future trials of interventional AF therapies should focus on patients with ischemic HF and ensure long‐term follow‐up.

## Sources of Funding

This work was supported by the British Heart Foundation (PG/08/020/24617). The research is supported by the National Institute for Health Research (NIHR) infrastructure at Leeds. The views expressed are those of the authors and not necessarily those of the National Health Service, the NIHR, or the Department of Health.

## Disclosures

L.K. has received speaker fees from Merck (modest), NovoNordisk (modest), and unrestricted research awards from Medtronic (modest). K.K. Witte has received speaker fees from Medtronic (modest), Livanova (modest), St. Jude Medical (modest), Pfizer (modest), Bayer (modest), and BMS (modest). The remaining authors have no disclosures to report.
